# Birthing the placenta: women’s decisions and experiences

**DOI:** 10.1186/s12884-019-2288-5

**Published:** 2019-04-27

**Authors:** Rachel Reed, Laura Gabriel, Lauren Kearney

**Affiliations:** School of Nursing, Midwifery and Paramedicine, University of the Sunshine Coast, Locked Bag 4, Maroochydore DC, Qld 4558 Australia

**Keywords:** Placenta, Third stage, Decision-making, Childbirth, Women’s experiences

## Abstract

**Background:**

Previous research examining the birth of the placenta has focussed on quantitative outcomes comparing active and expectant (physiological) management. However, it is also important to understand women’s experiences of birthing the placenta.

**Methods:**

The participant group consisted of 11 women who had expectant management, eight who had active management and one who was unsure. Participants were interviewed in-depth and the data analysed using thematic analysis.

**Results:**

Seven themes were identified in the data relating to before, during and after the birth of the placenta. Before birth themes focused on making decisions and included ‘doing the research’ and ‘natural birth’. During the birth of the placenta themes were ‘boundaries of time’, ‘focusing on baby’ and ‘sensations’. After the birth themes consisted of ‘looking’ and ‘keeping’.

**Conclusion:**

Most of the women considered a physiological birth of the placenta to be an intrinsic element of natural birth. Active management was considered to be an intervention used if complications occurred. In contrast, women who chose active management did not consider the placenta to be an important element of natural birth, and chose active management in order to prevent complications. Decisions about birthing the placenta were informed by Internet sources and previous personal experiences rather than care providers. During the birth of the placenta care providers managed the boundaries of time whilst women focused on their baby. The sensations women described were consistent across both types of management. Women valued seeing their placenta and having the opportunity to keep it, and placenta encapsulation was popular. The findings of this study contribute the experiences of women to the body of knowledge informing practice during the birth of the placenta.

## Background

This study examined women’s decisions about, and experiences of, birthing the placenta. A feminist framework underpinned the study and placed the perspective of women at the forefront. Therefore the language used throughout this article reflects a commitment to a woman-centred understanding of birthing the placenta. In medical literature the birth of the placenta is referred to as the ‘third stage of labour’ [1], a term that reflects the biomedical notion of clearly definable stages of labour. However, the concept of stages of labour does not reflect current understandings of physiology [2, 3] or women’s experiences of labour [4, 5]. Therefore, the term ‘third stage of labour’ has been replaced with ‘birth of the placenta’ or ‘placental birth’ throughout this article. This study focussed on birthing the placenta after a physiological birth. Physiological birth refers to a healthy labour that occurs without the need for medical or surgical intervention, and results in the uncomplicated vaginal birth of the baby [6].

Care providers commonly use either active management or expectant (physiological) management to facilitate the birth of the placenta. Active management traditionally involves three components: administration of a uterotonic medication; early clamping and cutting of the umbilical cord; and, controlled cord traction [7]. In recent years individual components of active management have been challenged, and recommendations have evolved in response to research evidence. In particular, the recommended timing of umbilical cord clamping has been extended to facilitate optimal blood transfer from placenta to baby [8]. Alternatively, expectant management involves the care provider supporting the woman to birth her placenta without intervention [9]. The role of the care provider during expectant management is to facilitate an environment that supports the physiological process whilst monitoring blood loss and the wellbeing of the mother [9]. Although expectant management increases the chance of postpartum haemorrhage (PPH) in all risk populations, less is known about outcomes relating to low risk populations. Some studies found that active management increased the chance of PPH in low risk populations [9–11]. However, the current World Health Organization guidelines recommend active management for all women to reduce the incidence of PPH [8].

To gain consent for management of the placenta, care providers are required to provide adequate information about the risks and benefits of both options [12]. However, women utilise a range of information sources to inform their choices about birth. In particular, the Internet enables women to access a wide range of information and facilitates connection with other women’s opinions and experiences [13, 14]. Women’s decisions are also heavily influenced by their own personal beliefs and attitudes, and previous experiences of birth [14]. Therefore, women may make decisions that do not align with clinical recommendations or their care provider’s preferences. Care providers are required to support women’s decisions even if they do not align with recommendations [15]. Disregarding or undermining women’s decisions can cause distress and trauma [16].

Previous research examining the birth of the placenta has focused on quantitative outcomes relating to care provider interventions [7]. To date, there has been no published research specifically examining women’s own experiences of birthing the placenta. There is also limited research examining women’s experiences of physiological birth in general. Most studies exploring women’s experience of birth include participants who had medical interventions [17–19]. These studies identify perception of control as an important factor in women’s emotional experience of birth and satisfaction with their care. Women report that they want to have a sense of control over external factors (what is done to them), and control over them selves (their own body and behaviours). The few studies that specifically explore women’s experience of physiological birth also identify that a sense of control over what is done by care providers is important [5, 20, 21]. However, during physiological birth women identify ‘letting go’ and ‘relinquishing control’ of the body/self as an intrinsic part of the birthing process. Whilst none of these studies specifically focused on the birth of the placenta, participants in Dixon et al.’s [21] study did provide some limited descriptions of birthing the placenta. In particular they described being focused on their baby rather than thinking about the birth of their placenta. Some considered birthing the placenta to be an ‘anti-climax’, whilst others considered it part of the recovery process.

In many countries, midwives provide the majority of care during birth for low risk women [22]. There is a growing body of evidence supporting physiological birth [2], and there is an expectation that midwives promote and support physiology [6]. Midwifery care should be woman-centred and be informed by women’s experiences [22]. However, to date there has been no research examining women’s experiences, perspectives or preferences regarding placental birth. This study is the first to specifically explore women’s experiences of birthing their placenta, and therefore makes an important contribution to the body of knowledge in this area.

## Methods

The study employed a qualitative interpretive approach that was underpinned by a feminist framework. Qualitative research allows an exploration of a phenomenon from the perspective of the person experiencing it [23], in this case, women’s experiences of birthing the placenta. In feminist research the primacy of women’s experiences is key, participants are placed at the centre of the inquiry, contributing directly to the generation of knowledge [24]. A feminist approach is particularly relevant to research about birth because birth is a female experience that takes place within a patriarchal maternity system [25]. It is important that women’s voices are listened to and valued when developing knowledge and practice that directly affects them.

Ethical approval was obtained by the University of the Sunshine Coast Human Research Ethics Committee (approval no. A/17/1004). Data were de-identified upon transcription and pseudonyms are used throughout this article to protect the identity of participants.

### Participant recruitment

Purposive sampling was used to recruit participants by disseminating invitations to participate via Facebook groups that engage new mothers. The Facebook posts invited women who had birthed in Australia to participate, and included information about the study, and the inclusion criteria. Women responded to the invite by emailing the research team to receive a Participant Information Sheet and Consent Form. If the woman met the inclusion criteria and consented to participation, an interview was arranged. The inclusion criteria required that participants were over 18 years of age and had experienced: a spontaneous labour without an epidural or narcotic analgesia; a non-instrumental vaginal birth; and an uncomplicated birth of the placenta in the last six months. In total 20 participants were recruited from Queensland, New South Wales, Victoria and Western Australia. The aim was to recruit 20 women but to continue recruiting if theoretical saturation did not occur during data analysis [23]. In addition, the aim was to obtain a sample of women whose experiences included active management of the placenta, and expectant management of the placenta. The participant details of the sample are reported in Table 1.

**Table 1:** Participant details

Pseudonym	Parity	Antenatal Decision	Type of Management	Care Provider	Birth Location	State
Annika	2	Active	Active	Private Obstetrician	Private Hospital	QLD
Brenda	3	Active	Active	Private Obstetrician	Private Hospital	QLD
Cynthia	1	Expectant	Expectant	Private Obstetrician	Private Hospital	QLD
Diana	2	Expectant	Active	Private Obstetrician	Private Hospital	VIC
Evelyn	1	Expectant	Expectant	Midwife (group practice)	Public Hospital	NSW
Fiona	2	Expectant	Expectant	Private Practice Midwife	Home	NSW
Gina	2	Expectant	Expectant	Private Practice Midwife	Home	QLD
Helen	2	Active	Expectant	None	Home (unplanned)	VIC
Ingrid	3	Expectant	Expectant	Midwife (group practice)	Public Hospital	QLD
Jessica	2	Expectant	Expectant	Private Practice Midwife	Home	NSW
Kate	2	Expectant	Expectant	Private Practice Midwife	Home	WA
Lisa	3	Expectant	Expectant	Hospital Midwife	Public Hospital	NSW
Maya	2	Expectant	Expectant	Midwife (group practice)	Public Hospital	QLD
Nicole	1	Expectant	Expectant	Private Practice Midwife	Home	NSW
Olive	1	Expectant	Unsure	Hospital Midwife	Public Hospital	NSW
Penny	1	‘Wait and see’	Active	Hospital Midwife	Public Hospital	QLD
Rebecca	2	Expectant	Active	Private Practice Midwife	Public Hospital	QLD
Sally	4	‘Wait and see’	Active	Midwife (group practice)	Public Hospital	QLD
Tanya	3	No choice	Active	Private Obstetrician	Private Hospital	NSW
Ursula	1	No choice	Active	Hospital Midwife	Public Hospital	WA

### Data collection

Semi-structured in-depth interviews were used to collect data. Each participant was interviewed via telephone or face to face (by author 2). The date and time of the interview was arranged to meet the preferences of the participants, and interviews lasted between 12 min and 21 min. Interviews were based around a number of semi-structured, open-ended questions to elicit information about participant’s individual experiences and perceptions of birthing the placenta (see Table 2). Whilst the focus of the study was not on the birth of the baby, women were invited to share the details of this experience. This enabled the interviewer to confirm the inclusion criteria regarding the birth. In addition, this reinforced the value of the individual woman’s birth experience and story, which aligns with a feminist framework. Interviews were audio recorded and transcribed verbatim.

**Table 2:** Interview questions

• What information did you have about birthing the placenta before and during your pregnancy? •Where did you get this information from? • Did you make any decisions about how you would birth the placenta before labour? • What were your decisions and why? • Can you give me an overview of your labour and birth up to the birth of your baby? • Tell me about what happened in the time between your baby being born and the placenta being born? • Tell me about your experience of birthing the placenta? • What did you do with your placenta afterwards?	

### Data analysis

Data was analysed using an interpretive thematic analysis approach [23]. First, author 1 listened to the audio recordings of interviews, and read and re-read the transcripts. This allowed immersion in the data and the identification of broad categories and variations within the data. During this phase initial ideas were discussed and verified with author 2 who had conducted the interviews. The broad categories were further analysed to derive subthemes and overarching themes. Themes consisted of issues, thoughts or ideas that appeared across the majority of transcripts. Data were organised in a Word document with relevant quotes listed under theme headings. Saturation occurred after the analysis of sixteen transcripts when no new themes were identified in the data, and adequate data had been collected to support the identified themes [23]. The additional four transcripts provided further data to support the identified themes. Themes and subthemes were reviewed and discussed by all members of the research team to ensure consistency in analysis and findings.

## Findings

An overview of the characteristics of participant experiences is presented in Table 3. Seven themes were identified in the data and are presented within a framework reflecting the sequence of the experience of birthing the placenta (see Table 4). ‘Before’ focuses on decision-making, and includes the themes ‘doing the research’ and ‘natural birth’. ‘During’ describes women’s experience of birthing their placenta and contains the themes ‘boundaries of time’, ‘focusing on baby’ and ‘sensations’. ‘After’ explores what women did with their placenta after birth, and includes the themes ‘looking’ and ‘keeping’. The themes are presented below with supporting data including the type of placental management (active or expectant) that the woman experienced.

**Table 3:** Characteristics of participant experiences

	No	%
Parity		
Primiparous	6	30
Multiparous	14	70
Primary care provider		
Private Practice Midwife	6	30
Private Obstetrician	5	25
Midwife Group Practice	4	20
Hospital Midwife	4	20
None	1	5
Discussion with care provider about active vs expectant management		
Yes	5	25
No	15	75
Antenatal decision		
Expectant	13	65
Active	3	15
“Wait and see”	2	10
No choice	2	10
Birth Location		
Public Hospital	9	45
Private Hospital	5	25
Home (planned)	5	25
Home (unplanned)	1	5
Type of management		
Active	8	40
Expectant	11	55
Unsure	1	5

**Table 4:** Themes

Before: decision making	During: birthing the placenta	After: the placenta
Doing the research: Women had little or no information from their care providers and sought information outside of the care provider relationship	Boundaries of time: Women were aware that there were expectations regarding how long they could wait for the placenta. Care providers managed the boundaries of time by intervening to facilitate the birth of the placenta.	Looking: Women valued the opportunity to look at their placenta. Care providers acted as gatekeepers to this experience.
Natural birth: Women who chose expectant management considered the birth of the placenta to be an intrinsic element of natural birth.	Focusing on baby: During the time between the birth of the baby and the birth of the placenta, women’s attention was on their baby rather than the placenta or their care providers.	Keeping: Seven women talked about keeping their placenta. Five of the women had their placenta encapsulated.
	Sensations: Birthing the placenta was described as easy, gentle and painless. In some cases care provider actions were described as uncomfortable and painful	

### Before: decision-making

Women made decisions about how they would like to birth their placenta during the antenatal period. The majority of women chose physiological management (13) with three choosing active management and two deciding to ‘wait and see’. Two of the women (Tanya and Ursula) reported that they did not think they had a choice.

#### Doing the research

Women did their own research about birthing the placenta rather than relying on information provided by care providers. Many of the women had little or no information from their care provider about their options for birthing the placenta:

Absolutely nothing… But now that you’ve asked the question, I am a little bit surprised that I really don’t recall with any of my three pregnancies being given any information or even presented with the fact that I had a choice about how the placenta was going to be delivered. (Tanya – active)

In seven cases, when care providers offered information it appeared to reflect their own preferences or usual practice rather than a discussion of options. For example, Annika’s obstetrician presented active management as a statement rather than an option:

At the 36-week appointment with the obstetrician it was asked “so are you going to have the GBS swab” and then straight after “and are we going to do active management”. So she stated it, it was more of a statement than a question and no extra information was given. (Annika - active)

Tanya felt that questioning her obstetrician’s preferences might disrupt their relationship, and it was important for her to feel liked:

…I would say the decision was made for me… But to be honest I didn’t have the guts to question my obstetrician about the possibility of doing it without the injection… I don’t know, I just didn’t want to… it sounds silly saying it but I didn’t want to kind of ruffle my obstetrician’s feathers at all. It just meant a lot to me to feel that I had a good relationship with him, and that he liked me as a patient. (Tanya - active)

In contrast, three care providers offered expectant management as their standard approach. Gina had a planned homebirth, and her private practice midwives (PPMs) did not discuss active management as an option.

I didn’t have much [information] before my pregnancy, but then in my pregnancy I was told to get the injection and have it in my fridge available… if the birth is OK and stuff, she shouldn’t have to use it… I don’t think she did go through much about the injection itself… I’m sure my midwife coached me a little bit, and had her views on it, but I don’t really recall the specific stuff. (Gina - expectant)

However, five women reported having discussions with their care providers about options. In particular those with continuity of midwifery care:

And I loved the experience… having the midwife was really, really cool. I felt like I could ask her anything and she did go into depth because she knew that I cared. So I felt very informed, I felt very prepared. (Sally - expectant)

Women’s main source of information for decision-making was from personal research. Women talked about doing their own research about options for birthing the placenta: *“… I did a lot of personal research just so I knew what was going on”* (Olive - unsure). The majority of the woman (13) sought information outside of the care provider relationship. Penny (active) believed that *“If you don’t go looking for it* [information]*, you probably wouldn’t get it”*, and Nicole said that:

…unless you are searching for an alternative to the mainstream, no I don’t really think that it’s that accessible for women… My impression is that it’s just the expectation that you know, we’ll just hand the control over to the doctors because they know what’s best. (Nicole – expectant)

Women used a number of sources to find information including books and articles, and independent childbirth classes. In particular, the Internet provided access to information and communities. Jessica (expectant) said: *“everything was from the internet.”* And Lisa utilised Facebook groups to find information:

Facebook groups, so not really just in the group, but they would have links in there to articles, and that kind of thing mostly. (Lisa – expectant)

Women also sought out the experiences of other women, and the Internet facilitated this sharing of experiences and opinions:

I also was part of a few pregnant mums Facebook groups, and you’d ask questions in there and people would give their opinions. (Evelyn – expectant)

Other women’s experiences served as a warning for some. For example, stories of negative experiences with routine active management reinforced the decision to have expectant management:

…So I’d read heaps of stories, and personal accounts like that [intervention resulting in complications], that just made me think OK, fair enough if one person has a personal account like that, but the fact that I’m reading story after story, this is just happening too much. And I didn’t realise at the time that that is just what happens in hospital, you just… that that is how it is. (Fiona - expectant)

For Brenda, her friend’s experience of haemorrhaging reinforced her decision for active management:

That was one of my biggest fears this time was actually haemorrhaging. I don’t know why. I haven’t had the experience of it but I’ve had friends who have, and a friend who haemorrhaged… And I think because of that it was really in my mind. I think that’s where my fear came from. (Brenda - active)

Women also used knowledge gained through their own previous experiences to base their decisions on. In particular, four women chose expectant management after a previous birth experience:

I think for me the biggest thing was the lack of informed consent. They didn’t explain what the injection was or, you know what could go wrong if it didn’t work like it was meant to, and the fact that she was like ‘yep, give you this, alright you need to cough for us’ while she’s pulling on the cord. It was just very medical, and I guess not how I’d pictured it. (Kate – expectant)

#### Natural birth

All of the women in the study had uncomplicated physiological births of their baby. The women used the term ‘natural birth’ to describe this type of birth. Women who initially chose expectant management of the placenta considered the birth of the placenta to be an intrinsic element of natural birth. Physiological birth of the placenta was considered the ‘standard’ following a physiological birth of the baby:

I was hoping that if it all went smoothly then I would just go with the physiological because that’s I guess the standard, the normal, like what would have happened. (Lisa – expectant)

For these women, non-intervention throughout the entire birth experience was important:

I think it’s important, yeah, it was very important to me. I think it really completes that whole birth process. I think it’s really significant that it comes away on it’s own and it really completes the entire birth... So yeah, I thought it was really important to just let it happen as natural as possible, as it would with no intervention. I didn’t feel that it needs any intervention. (Ingrid - expectant)

In contrast, women who either chose active management or to ‘wait and see’ did not consider physiological birth of the placenta as an intrinsic element of a natural birth:

I definitely wanted to have as natural a birth as possible. So not having any injections would make it more natural, but I also was thinking ‘I’ll just wait an see’ and that part being natural was not as important as the earlier part being natural… For me it came as secondary to the baby… it just wasn’t as important as being drug free beforehand. (Penny - active)

Women who chose expectant management talked about trusting their bodies to birth naturally:

I just trusted my body, that was how it was meant to be, and I guess I didn’t ever think that my body couldn’t birth the placenta naturally… And even I don’t even know the side effects of the injection, for myself. I just chose not to… I just trusted my body to do it naturally the way it’s designed to do kind of thing. (Gina – expectant)

In two cases midwives fostered and reinforced this trust of the body for women:

I really liked the way it was worded to me early on, which was that, not to look at pregnancy as an illness, to look at it as just another state of being. And that saying really helped me through the whole process, and then just to… that second question ‘if we trust you enough to birth your own baby, why would we not trust you to birth your own placenta’ kind of thing. (Evelyn – expectant)

None of the women who chose expectant management suggested that they would not accept medical intervention if a problem arose. They considered active management to be something that they would have only if necessary, if birth was not physiological or a complication occurred. Active management was associated with a deviation from natural birth. For example, Kate stated: *“I knew that if needed, we could move onto the interventions, rather than start with them.”*

In contrast, both of the women who chose active management in the antenatal period expressed concerns about safety and risk in relation to physiological management. For them active management aimed to prevent complications rather than manage them. For example, Brenda considered active management to be the ‘safe route’:

And I think I was just a bit scared too, to try for anything different than what somebody recommended because you may as well just take the safe route and have the injection… (Brenda - active)

### During: birthing the placenta

Out of the 13 women who planned expectant management, 10 had expectant management, two had active management, and one was unsure what kind of management she had. Out of the three women who chose active, two had active management, and one had an unplanned birth of her baby and placenta at home, and was later given an oxytocic injection on admission to the hospital. The women who planned to ‘wait and see’ (two) or did not think they had a choice (two) all had active management.

#### Boundaries of time

Women were aware that there were expectations and deadlines regarding how long they could wait for the placenta. Both of the women who chose active management in the antenatal period wanted to limit the waiting time. Annika decided to have active management because she *“didn’t really want to be waiting around”* and *“just wanted it over and done with quickly.”* Brenda stated:

I think to me, once the baby is out I just want it over with. I’m a bit impatient like that [laughter]. I didn’t want to mess around with it. Yeah, just get it over with… (Brenda - active)

Women who had expectant management in a hospital setting were aware that there were time constraints about how long they would wait. Cynthia was able to have expectant management because she birthed her placenta within the agreed timeframe: *“because we had agreed that we would go no longer than, I think it was half an hour… so I didn’t have to make that decision.”* Diana initially chose expectant management but changed her mind while she was waiting for her placenta. She was worried that if her placenta took too long she would have to go to theatre:

I just remember waiting around after for the placenta to come out… I was thinking it was taking so long, like ‘oh my god, please come out placenta because I don’t want to have to go to theatre after this amazing wonderful birth’. (Diana - active)

Women also reported that their care providers were concerned about timeframes during expectant management. Evelyn’s midwives *“were worried that it was taking a little bit longer than anticipated.”* However, Evelyn herself *“wasn’t really worried about it.”* Lisa was aware that there was pressure on her midwife from the obstetrician outside of the room:

And the midwife was sort of in and out of the room. And then when we got to the hour she said… ‘oh the obstetrician is getting a little bit antsy that the placenta hasn’t come yet.’… I felt like she was holding the obstetrician off. So she was willing to keep going and trying a few things knowing that the physiological was what I wanted… I got the impression that she was sort of stretching it by waiting as long as we did as well. (Lisa – expectant)

Expected timeframes appeared to be different in a home setting. Women who had expectant management at home reported a lack of urgency, and their midwives demonstrated patience and trust in the woman’s ability to birth her placenta:

My midwife was just nice and calm and relaxed, and like I said, I actually forgot about it completely… she was just great with keeping everything calm I guess… My midwife was supportive of giving me more time than would necessarily be allowed in a hospital… I was pleased because ultimately it was closer to two hours before it came out, but it did all happen naturally and normally. (Jessica – expectant)

Nicole (expectant) described her experience as *“…incredible, I didn’t feel rushed or anything.”* When Gina became anxious her midwife reassured her:

I started to say ‘I just want it out, why is it taking so long?’ And she wasn’t worried at all, she’s like ‘It’ll come’. (Gina – expectant)

Care providers managed the boundaries of time by intervening to facilitate the birth of the placenta. The methods they used differed according to the birth setting. For example, hospital care providers suggested active management to ensure time deadlines were met. Penny was willing to accept this change of plan when it was offered:

They said ‘we need to get the placenta out’ and ‘do you want the injection or not?’ And I think I just went ‘yeah, just do it.’ I was just over it by then. (Penny - active)

However, Rebecca wanted to continue waiting:

I kind of wanted to just have a go at doing it… I think if it [injection] hadn’t been offered to me I would have just progressed to try and do it… like it wasn’t something that I was really thinking about. (Rebecca - active)

Care providers also pulled on the umbilical cord during expectant management to speed up the birth of the placenta. Lisa’s midwife *“had a little look and then a really gentle tug on the cord just to see if there was any movement”.* Maya’s midwife *“kind of gave the cord a little tug and it all just came out then.”* Cynthia’s obstetrician used uterine massage to deliver the placenta during expectant management:

…and the obstetrician was just sort of feeling around on my tummy and she said ‘oh I think it is about to come out’… She just sort of pushed on my tummy a bit, and yeah it came out. (Cynthia - expectant)

Midwives in the home setting also intervened to manage boundaries of time during expectant management. However, they used different approaches. For example, Jessica’s midwife used acupressure:

…after about the hour and a half she asked me if I felt any more contractions… and then she just asked if she could massage my legs, and I remember thinking ‘oh that’s really nice for her to offer to massage my legs’. But obviously she was just trying to do some acupressure there to try and help the placenta come out. (Jessica - expectant)

At homebirths midwives encouraged women into upright positions to use gravity to facilitate the birth of the placenta:

“… after a little while my midwife suggested standing up and seeing if gravity could help. Basically as soon as I stood up the placenta came out…” (Kate - expectant).

In particular, homebirth midwives encouraged women to sit on the toilet while waiting for the placenta to birth:

…she [midwife] said ‘look, do you want to just come and sit on the toilet, maybe gravity will just help it come out’. And so, I just walked over to the bathroom, and she put a bowl in the toilet, and I sat on the toilet and we were just having a chat in there, and actually looking through some of the photos that they’d taken of the birth. (Jessica – expectant)

Four midwives left women alone in the toilet to facilitate privacy. Jessica (expectant) birthed her placenta after her midwife left the bathroom. Her midwife told her: *“‘that happens with so many women’, she said ‘I just think placentas are shy’* [laughter].”

#### Focusing on baby

During the time between the birth of the baby and the birth of the placenta, women’s attention was on their baby rather than the placenta or their care providers. Jessica *“completely forgot about the placenta, it was not on my radar at all”*, and Diana said that:

…I really didn’t have time to think about the placenta because I felt so busy just thinking about my baby and being excited that I’d given birth to her and enjoying that moment with my family. Even, like, I don’t remember seeing my obstetrician or the midwife, I was just so enthralled… in this moment with my baby and my husband and my sister. (Diana - active)

Women described how focusing on their baby distracted them from taking in what was going on around, and to them. Brenda (active) found it difficult to remember what happened because she *“was just too distracted by her* [baby].*”*

Three of the women described being separated from their baby while waiting for the placenta to birth. Two of the women having expectant management in hospital experienced separation from their baby initiated by their care provider:

My partner had the baby at that point and it was the obstetrician and the midwife both trying to… It was like ‘oh [name], bub’s dad will take him while we sort of just get this done’. (Cynthia - expectant)

Sally was the only participant who requested that her baby was removed from her so that she could focus on birthing the placenta. However, this was after her midwives started uterine massage:

I wasn’t really feeling contractions for the placenta again. And so, that’s when [midwife] and I had a student midwife, so they came back over and they started to sort of, you know push down on the top [uterine massage]. I started to decline in motivation quite quickly, and I started to feel really anxious. I wanted the baby away from me, like I needed to focus on the placental delivery. And I had the baby taken away, like my husband held her, and then it just got really, really full on. (Sally – active)

#### Sensations

Women described the physical sensations of birthing the placenta, and most described their experience of the placenta coming out as ‘easy’, ‘gentle’, and ‘painless’. For example, Ursula (active) said: *“…it felt like it was quite a gentle process”*, and Ingrid described her experiences of birthing her placenta as:

I’ve never experienced that to be painful or uncomfortable, when it’s birthed. I’ve always found that quite an easy process to birth the placenta… there’s no discomfort at all when it is birthed... (Ingrid – expectant)

Some women described feeling cramps and pressure as the placenta birthed, however, they did not consider these sensations painful. Women having active, and women having expectant management described the same sensations:

I just remember having a little bit of cramping in my tummy, not as strong as when I was giving birth, but a bit of a cramp again, and sort of a bit of pressure down in the vagina… and feeling a bit of gushing blood, and then the placenta sort of came out after that like a bit of cramping in my belly. (Diana - active)

Only Sally described birthing the placenta as uncomfortable and painful:

I could tell that the next phase of labour needed to take place, and I started to become uncomfortable physically. Like I was in pain. It was almost like… I’ve had three babies, so this was like, ‘oh I know what’s coming, I hate this part.’ (Sally - active)

As mentioned above Sally’s midwife carried out uterine massage on her. Other women also described the actions of their care providers, in particular uterine massage, as uncomfortable and painful:

…massaging the stomach to get the uterus to contract. That was awful, yeah, that was very painful. (Penny - active)

Pushing up and like down… like fingers kind of going in and pushing around… Just lots of pressure. I think pushing on my tummy was really uncomfortable… I definitely used the breathing techniques to breathe through that… But, yeah it was quite hard to relax. (Brenda - active)

Women used terms such as ‘strange’, ‘weird’, ‘odd’ and ‘funny’ to describe the sensations of birthing the placenta:

The actual birth of the placenta was just weird, just because it was so soft. It felt like my insides were coming out but not in a painful way. I guess it’s like birthing a squid or something [laughter]. But no, definitely not uncomfortable, it was more just a strange sensation. I guess maybe like sort of pulling on a tampon a little bit. (Nicole – expectant)

The placenta was described as ‘soft’ and ‘warm’ and women talked about what it felt like as the placenta birthed using terms such as ‘plop’, ‘slid’ and ‘fell’. Rebecca (active) described how her placenta *“pretty much just slid out”*, and Maya used the term ‘wobbled:’

I do remember feeling a slimy large thing coming out. I definitely felt it. I don’t know how you would even describe it… I said ‘it plopped out’, but yeah it kind of just wobbled out. I could feel it passing through my vagina but I couldn’t feel any pain or discomfort or anything like that. It was just a sensation. (Maya – expectant)

Women also referred to the experience of birth the placenta as ‘nice’:

…compared to birthing a baby, it’s like birthing a big thing of jelly [laughter]. It’s quite soft and a bit sort of… pushing a placenta out compared to a baby, it’s almost like a nice little ‘oh, that was nice’ [laughter]. (Rebecca - active)

Only Olive described the experience in negative terms:

…I honestly felt that it was more uncomfortable than the birth. It just felt disgusting, and I know that sounds really bizzare. Like you know something’s coming that you want, but yeah it just felt really, really uncomfortable… but no pain in anyway, just a little bit of discomfort. It was just a very odd feeling in that way. (Olive - unsure)

Many of the women described a sense of relief – both physical and emotional – once their placenta was born. Lisa (expectant) said *“pushing it out felt like a big jelly, relief”*, and Diana (active) described feeling *“a release and relief…”* The birth of the placenta appeared to signal the end of the birth process, and women felt a relief that it was over and they were well:

Yeah sort of a bit relieved then because it was a recognition of like ‘ok all of that’s done’. And yeah it kind of a nice feeling… (Cynthia – expectant)

### After: the placenta

#### Looking

Women talked about looking at their placentas; and on the whole women wanted to see their placentas. Sally (active) stated that she was *“keen to look at it”*, and Maya (expectant) said she *“was really interested to have a look.”* Only two women (Olive and Rebecca) said that they did not want to see their placenta. Eight women described how their care providers acted as gatekeepers to the experience of looking at the placenta. Four women talked about how their midwives asked if they would like to look at their placenta, and then displayed and explained the placenta for them:

…so she laid it out, she put a mat down on the floor, and the surgical… whatever it is… and she showed it to me, and she showed me the inside of it and explained what she’s looking for is to make sure that it’s all intact, and she showed me that it was all intact. And then she showed me… she pulled up the sac on the outside and showed me how that’s what holds the baby in there when they are inside. She showed me the actual cord and the three tubes inside, how baby gets nutrients and all that kind of stuff. And then, yeah we took some photos of it… (Jessica - expectant)

Other women (four) reported that their providers did not offer this opportunity. Tanya asked about her placenta but her obstetrician did not show her:

…I kind of lifted my head and said ‘oh how does it look, and is it healthy?’ and was intrigued to have a look. And I just remember seeing a mass of red, bloodied organ looking stuff between my legs and it being slopped into a stainless steel tray and taken away. (Tanya – active)

Diana and Evelyn expressed regret that they did not get to see their placentas:

...I’m not even sure what they did with it. I didn’t actually look at it this time… None of the practitioners asked me if I wanted to see my placenta. I think that is an important thing to offer. Because if you’re not offered, you don’t think about it as a woman. (Diana - active)

Unfortunately not, no, I didn’t have a look at it. That’s probably one of my regrets from the birth is not to have seen what it looks like… I didn’t have a look at it. I wish I had. (Evelyn – expectant)

The women who did see their placentas described them as ‘interesting’ and ‘fascinating’. For example Sally (active) said her placenta was *“really cool, really interesting.”* Maya reflected that *“it would be nicer if it was kind of honoured a bit more.”* Other women also talked about the importance of the placenta:

After I saw [baby’s] placenta, and just how I realized that they’re all unique. Like every one’s placenta, each person, each baby, they are all different. And yeah, I do find them a little bit fascinating. I’m not a huge body organ fan either, but I do respect them as an organ, and I do think it’s really cool. So, it was an important part for me to see it because part of growing my baby, was also growing the placenta, so it was something that I respect. (Gina - expectant)

Only Ursula (active) described her placenta in negative terms, stating that *“…it just looked like liver so I was just ‘it looks gross either way to me’”*.

#### Keeping

Most of the women either did not talk about keeping their placenta (seven), or specifically stated that they did not keep their placenta (six). The reasons given for not keeping the placenta appeared to be about a lack of planning or logistics. For example, Ursula’s (active) placenta was kept by the hospital because *“they believed something weird was going on with it, they sent if for testing.”* Kate (expectant) *“thought planting it could have been a bit risky”* because she lives in the bush, so instead the placenta was disposed of by the hospital. Penny simply *“didn’t want to keep it”*, and Sally (active) said that she *“didn’t keep it because I hadn’t organised anything, so I didn’t know what I’d do with it.”* She went on to say:

…but I do wish I had a bit more information about what to do with the placenta afterwards. I felt like it was a bit of a shame to just see it go in the bin. And I don’t think that there is any procedure behind that, and I understand that, but I think I would have… I think it was kind of an after thought and I wish I’d done a bit more preparation on that. (Sally – active)

Seven women stated that they kept their placenta. Evelyn planted a tree over her placenta:

My mum did [plant a tree], with ours, so I just thought it was a nice sentimental sort of thing. I’m a sentimental person so I thought it would be a nice thing to do. (Evelyn – expectant)

Diana and Gina’s midwives made placenta prints for them. Gina described how her *“midwife painted it and then printed onto a piece of paper.”* Diana also talked about what her midwives did with her placenta:

They did a placenta picture, and I’ve actually got that framed in my daughter’s bedroom. It might be a bit corny [laughter]. And they dried out a cord stump as well, like a bit longer than the one that falls off the baby… And I also really liked that as part of that process too, that the woman did a placenta print. Because I wanted to do that with my first son’s placenta myself, but my husband was a bit ‘eeer, that’s gross’. (Gina – expectant)

Five of the women had their placenta encapsulated. Their reasons for doing this centred around perceived health benefits:

…one of the midwives who looked after me does the encapsulation process. So she discusses that a lot with her women… I have a lot of hormonal migraines, so that was one of the big reasons that I wanted to try it. But I just found that it did help hormonally, I didn’t feel quite so up and down in those first few months with my breastfeeding and return to my menstrual cycle, and all that sort of thing. I sort of felt like I wasn’t quite so up and down like I had been with my previous babies. (Ingrid – expectant)

Nicole’s midwife also offered an encapsulation service:

I think I’d done a bit of research and a bit of reading and I knew that [midwife] did it and she sort of said for some women it does nothing, but for other women it can help regulate mood, it can help stabilize hormones, it can help with milk production. And for me, it just kind of seemed like a natural progression…And it was actually a really great experience. [Midwife] said I might feel a bit manic, if I feel too energetic to back it off a little bit. But… I didn’t have any really deep lows. I never felt overwhelmed with sadness or shock of having a baby. I always had enough energy despite looking after a newborn. And I don’t know if that was just hormones anyway but I do think that the placenta pills had something to do with that. (Nicole – expectant)

There was also a sense of making the most of the placenta by encapsulating it:

Yeah I got it encapsulated for the health benefits… Better than just throwing it away I thought. And I’d heard as well that you know we are the only mammals that throw our placentas in the bin and that also really stuck with me when I was pregnant, someone said that, and I was like ‘yeah that doesn’t make sense.’ If everyone else in the wild is doing something with their placenta then maybe we should as well so, yeah it was important for me to not just chuck it away and disregard it. (Gina – expectant)

Diana felt encapsulation was a way to keep her placenta:

… this time I encapsulated my placenta, but last time I didn’t so I really felt that the placenta was just taken away from me, and never to be seen again. Whereas I think this time, because I encapsulated it, I felt like I still had it… I also wanted to do it so that I could keep it, like just not in it’s original form but have some of it in a form I could keep. (Diana – active)

Evelyn and Ursula considered encapsulation but decided against it. Brenda and Maya had encapsulated their placenta in a previous pregnancy, but chose not to again. Brenda (active) said that this time she *“couldn’t be bothered. And I don’t know if it actually helped.”* Maya chose not to this time because she felt well after the birth:

…my first birth didn’t go so well so I was feeling quite rubbish afterwards so I wanted to maybe encapsulate my placenta. [With this birth] I didn’t, I had such an easy birth that I actually felt fine afterwards so I didn’t do it. (Maya – expectant)

Out of the 20 participants, nine had either experience of placenta encapsulation (five with this birth, two with a previous birth), or had considered this as an option (two).

## Discussion

All of the women in this study wanted a ‘natural’ birth regardless of what type of management they chose for the placenta; and these findings align with a systematic qualitative review that found most women aimed to have a physiological labour and birth [19]. However, women’s ideas about the importance of placental birth to the achievement of a natural birth varied. Women who chose expectant management considered a physiological birth of the placenta as an intrinsic element of a natural birth. They trusted their bodies to complete the birthing process without intervention, whilst also being open to active management if complications arose. For these women, active management was considered an intervention to be carried out in response to a complication. In contrast, women who chose active management did not consider how the placenta was birthed to be important for a natural birth. They were aware of potential complications, and were less trusting of their body’s ability to birth the placenta naturally. Active management was considered to be the ‘safe route’, to prevent complications, and thereby avoid further interventions. This approach is aligned with the current recommendations and practice [8]. However, there is increasing evidence that for women who have a physiological birth of their baby, active management may increase rather than decrease the risk of complications [9–11].

Care providers are responsible for providing adequate information to women in order to facilitate and support their decision-making [8, 12]. However, in this study most women (no = 15) reported that their care providers did not discuss the risks and benefits of active versus expectant management with them. Care providers appeared to share information that reflected their usual management, for example, obstetricians recommended active management whereas homebirth PPMs assumed they would carry out expectant management. It can be argued that this reflects the differences in experience and practice between the two professions rather than the preferences of women [26]. Whilst guidelines recommend active management for all women, they also maintain that women’s preference for expectant management should be supported for women at low risk of PPH [27, 28]. For women to express a preference, they require information about all options. However, research has demonstrated that care providers often fail to assist women in making informed decisions about maternity care [29, 30]. In this study women also suggested that care providers in general could not be relied on to provide adequate information.

Women in this study mostly sourced information for decision-making outside of their care provider relationship; and the Internet was a primary source of information about options for birth. Other studies have also identified the Internet as an important, and effective source of information for women’s decision-making about birth [13, 31]. In this study, the Internet provided access to information and communities, and importantly to other women’s experiences and opinions, which reinforced and supported decision-making. These findings support those of a meta-synthesis by Sanders et al. [14] exploring the impact of information sources on women’s decision-making for birth. The meta-synthesis found that women distrusted care provider’s ability to facilitate informed decision making, which fuelled women’s need for information and connection outside of the care provider relationship. The Internet offered a wider range of views and enabled access to information that confirmed and justified women’s own philosophical approach and preferences. It also facilitated connectivity with other women, and access to a range of experiences and opinions. Many women in the meta-synthesis reported that information gathering provided a sense of control and empowerment. Therefore, it can be argued that care providers should encourage and support this process. Lagan et al. [31] maintain that care providers need to acknowledge that women use the Internet for support and actively engage them in discussion about the information they access.

Sanders et al. [14] also found that women drew on their own embodied experiences when making decisions, and some aimed to avoid re-experiencing negative aspects of a previous birth. Women in this study also based their decisions about the birth of the placenta on previous experiences. In particular, four women chose expectant management after a previous experience of medical intervention during birth. Understanding how women access information and make decisions can assist care providers to meet their needs. For example, by exploring the woman’s own philosophical position, previous experiences and any information she has gathered. Institutional recommendations need to be discussed in relation to the woman’s individual circumstances and preferences to facilitate informed decision-making.

Clinical guidelines define a prolonged birth of the placenta as 30 min for active management and 60 min for expectant management [27, 28]. However, no research is cited to support these specific time limits. PPH is associated with a longer duration between the birth of the baby and the birth of the placenta during active management [32]. Further research is needed to inform timeframes for expectant management. Women in this study were aware that there were time constraints around birthing the placenta regardless of their chosen management. They also reported that care providers were concerned about time, and often intervened to facilitate the birth of the placenta during expectant management. However, the time parameters and interventions used varied according to birth setting. For example, women experiencing expectant management at home reported a lack of urgency from their midwives. To facilitate the birth of the placenta midwives encouraged positional change and privacy to augment physiology. In a hospital setting care providers were more likely to implement active management if expectant management exceeded the prescribed timeframes. In this study five women had expectant management in a hospital setting. However, four of these five women experienced controlled cord traction or uterine massage during their expectant management. Neither of these interventions are consistent with expectant management guidelines [9, 27, 28].

While waiting for the placenta to be born, women described being focussed on their baby rather than on what was going on around them; and these findings are consistent with the findings of Dixon et al.’s study [21]. Physical contact and interaction with the baby after birth facilitates the release of oxytocin and supports physiological birth of the placenta and reduces the chance of PPH [33]. However care providers cut the umbilical cord and removed the babies of two participants during expectant management. This practice is not recommended during expectant management and may increase the chance of PPH [9, 27, 28]. Care providers working in settings where active management is the norm may benefit from education and support to develop their practice of expectant management.

This study also captured women’s descriptions of their feelings and sensations during the birth of the placenta. Women used terms such as ‘strange’ and ‘weird’ to describe their sensations, and described the placenta as feeling soft and jelly like. When talking about the placenta being born women used words such as ‘plop’ and ‘slid’ suggesting a lack of effort. Although women experienced uterine cramping, they described birthing the placenta as painless and used terms such as ‘gentle’. Descriptions were consistent between women who experienced active and expectant management. These findings align with a study by Jangsten et al. [34] who found that women’s experience of pain during or after the birth of the placenta was not influenced by the type of management they had. However, in this study three women experienced care provider actions as painful, in particular uterine massage. Uterine massage is not recommended during uncomplicated active or expectant management [8]. Once the birth of the placenta was completed women reported a sense of physical and emotional relief. These findings can help to inform information sharing with women about what to expect during the birth of the placenta. In addition, care providers need to consider how women experience interventions such as uterine massage.

Women in this study described looked at their placenta, and care providers acted as gatekeepers to this experience by offering to display the placenta, or in some cases not (four). Women found their placentas ‘interesting’ and ‘fascinating’ and considered the placenta to be an important organ that deserved attention. The two women in the study who did not get to see their placenta expressed regret. Burns et al. [35] also found that women birthing in hospital expressed a sense of loss at not seeing their placenta. Current guidelines do not include recommendations for care providers about offering to show the placenta to women. However, guidelines do acknowledge that some women may request to take the placenta home, and offer recommendations for its transport and storage [28]. Burns et al. [35] explored placenta rituals amongst Australian women and identified that women who birthed outside of hospital were more likely to keep their placenta and consider the organ meaningful beyond its physiological function during pregnancy. In this study women who birthed in hospital also talked about the importance of seeing and keeping the placenta. However, only women who planned expectant management kept their placentas.

Seven of the women in this study had experience of placenta encapsulation. These findings reflect an increase of placentophagy in well-resourced countries including Australia [36, 37]. Selander et al. [38] carried out a survey of self-reported motivations and experiences associated with placenta consumption in the United States and Canada. They found that most women (80%), like the women in this study, ingested their placenta after it has been steamed, dehydrated and encapsulated rather than raw. Women perceived a number of health benefits from consuming the placenta including improved mood, decreased fatigue, balancing of hormones, improvement of lactation and increased iron levels. These findings are consistent with this study’s findings identifying the reasons for encapsulating the placenta centred around the perceived health benefits of placentophagy. However, the effects and safety of placentophagy remain unclear as studies have not been yet been carried out examining human placentophagy [36, 37]. In addition, concerns have been raised about the consumption of bacteria and other elements within the placenta [37, 39]. Despite the lack of evidence, encapsulation services are increasingly available, and in some cases offered as an additional service by midwives. However, in an online survey Cremer and Low [40] found that respondents were mainly aware of placentophagy through media, online and friends, with only 2.3% reporting that the information came from health professionals. These findings suggest that the increase in encapsulation services may be meeting consumer demand rather than driving it. Considering the popularity of encapsulation, further research is required on this topic to inform women’s decision-making and care provider practice.

## Limitations

This study is qualitative; therefore the findings are specific to the context of the study. The sample consisted of women who had a physiological birth without intervention. However, in Australia only a minority of women experience this type of birth [41]. In addition, most of the participants wanted expectant management of their placenta. This may indicate that these women were well informed and motivated regarding their birth choices. The participants in this study may not representative of the majority of women birthing in Australia.

## Conclusion

This study is unique because it focuses on the birth of the placenta from the perspective of women. Most of the women in the study considered a physiological birth of the placenta to be an intrinsic element of natural birth. Expectant management was the default, and active management was an intervention to be used if a complication arose. In contrast, women who chose active management did not consider the placenta to be an important element of natural birth; and they chose active management in order to prevent complications. In general women did not trust care providers to provide adequate information about options for birthing the placenta, and instead, they did their own research. During the birth of the placenta women focused on their babies rather than what was going on around them. However, they were aware of time constraints and their care providers managed the boundaries of time by intervening to facilitate the birth of the placenta during expectant management. The sensations women described were consistent across both types of management. Birthing the placenta was not painful, but uterine massage carried out by care providers was. Women valued seeing their placenta and having the opportunity to keep it. Placenta encapsulation was popular, and placentophagy was considered to confer health benefits. The findings of this study provide further information about women’s experience of birthing the placenta. In combination with research findings about outcomes, these findings can inform care provider’s discussions with women about their options, and inform practice during the birth of the placenta.

## Abbreviation

PPH: Postpartum haemorrhage
